# Bibliometric Analysis of Post Covid-19 Management Strategies and Policies in Hospitality and Tourism

**DOI:** 10.3389/fpsyg.2021.769760

**Published:** 2021-11-15

**Authors:** Kanwal Iqbal Khan, Adeel Nasir, Saima Saleem

**Affiliations:** ^1^Institute of Business and Management, University of Engineering and Technology, Lahore, Pakistan; ^2^Department of Management Sciences, Lahore College for Women University, Lahore, Pakistan; ^3^Institute of Quality and Technology Management, University of the Punjab, Lahore, Pakistan

**Keywords:** bibliometric analysis, management strategies (MS), COVID-19, hospitality, tourism, post Covid

## Abstract

The strategic perspective of management policies gained utmost importance during the post-Covid era. The researchers are trying to introduce strategies that can help organizations cope with post-crisis destruction. Yet, the research on the topic is fragmented, mainly related to the hospitality and tourism industry. This manuscript aims to present scholarly research findings dealing with the post-Covid-19 management strategies in the hospitality and tourism industry from January 1, 2020, to July 28, 2021. These strategies can play an essential role in the survival and growth of the sectors. The study identified and acknowledged the core contributing authors, journals, countries, affiliation, corresponding authors through bibliometric, citation, and keyword analysis. It also conducted the co-occurrence analysis and reported three significant research streams and bibliometric coupling to identify four research themes for management strategies of the tourism and hospitality industry in the post-Covid era. With the help of an influential and conceptual framework, the study highlights the future challenges managers could face and suggests the possible area for reviewing and revising the existing policies by proposing future directions. Consequently, this study contributes to the current literature on post-Covid-19 management strategies and policies by developing the critical analysis of the extant literature and highlighting the understudy areas that future studies must explore to expand the scope of the research.

## Introduction

Covid-19 becomes an unprecedented world challenge for the organizations that impact core business activities nationally and internationally (Ibn-Mohammed et al., 2021). Although it adversely affects all the segments of the economy, but the hospitality and tourism industry has been acutely affected by it (Casado-Aranda et al., 2021). The national lockdown policy and international travel restrictions are the main reasons for the cancelation of economic activities, severely affecting the hospitality and tourism industry (Khan et al., 2021). However, earlier to this pandemic, these industries were flourishing globally. Still, due to this worst-case scenario, many companies face a financial disaster that either leads to bankruptcy or leaves no choice to adopt a cut-off cost policy. This situation influences the firm’s financial policy and creates chaos among the employees regarding their future employability. In order to survive from the severe pandemic crisis, organizations wanted to establish post-Covid management strategies that would help them cope with the situation.

Scholars are publishing a significant number of articles to device solutions to the problem and evaluating the consequences of the pandemic for the hospitality and tourism industry. For that purpose, there is a need to conduct a bibliometric analysis to synthesize the findings of a large number of scholars in the field. Specifically, they are more interested in the two issues: first in future solutions of the crisis through better management and second in a quick recovery of the sector. At the same time, most of the attention is paid to the recovery process. Dastgerdi et al. (2021) emphasized introducing effective development plans that guarantee a healthy environment for tourists and provide a safe destination by controlling over-tourism. Del Buono et al. (2020) reported tourist flows and social mobility as the potential reasons for the Covid-19 outbreak in Italy. A study by Luca et al. (2020) highlighted the neglected dimensions in the management strategy: climate change, environmental concerns, crisis management, and cultural development plans.

The hospitality industry is switching from traditional to innovative business models to facilitate their customers (Marasco et al., 2018). Companies started innovative training programmes to equip their employees with knowledge-based skills that increase the customers’ revisit intention (Gupta and Sahu, 2021). The visitors prefer the hotels that strictly follow the SOPs for Covid-19, maintain stringent hygiene, and show extra care for the guests during service delivery. All these can be possible if companies implement innovative programmes, specially Covid-19 awareness and environment management training for their employees (Ebersberger et al., 2021). The companies are also adopting artificial intelligence tools and robotics technology to reduce human interaction under this pandemic situation (Yang et al., 2019). Although some ethical and legal issues are linked with its application, researchers and practitioners are recently finding its solutions (Fusté-Forné and Jamal, 2021). Another management related strategy that scholars emphasized to adopt to deal with post-pandemic challenges is applying green practices. Yousaf et al. (2021) stated that the hospitality industry could achieve sustainable development through green business strategies. Therefore, organizations should implement eco-friendly practices to keep the environment safe.

Although the researchers discussed the post-Covid strategies relevant to the field. But still, no comprehensive review has been conducted to report the growth of scholarly publications in the hospitality and tourism industry that identified the main management strategies and policies to handle the consequences of a virus outbreak effectively in the future. This approach helps to specify the current advancement in the field that help to reveal the strategic perspective and behavioral changes that occurred in these sectors. In light of the arguments mentioned earlier, the present study aims to: (1) find out the growth of publications on the post-Covid management strategies in the hospitality and tourism sector, indexed in the Scopus database between January 1, 2020, to July 28, 2021; (2) identify the core contributors (journals, authors, institutions, and countries, etc.) that are worth considering for devising the future management strategies; (3) suggest the key emerging themes and research streams for the prospective stakeholders; and (4) help to develop pandemic resistance strategies that provide future solutions to the unexpected setbacks.

The first two research objectives are academically essential to understanding the directions of hospitality and tourism literature after the pandemic. The third objective is intended to provide an in-depth insight into the main research streams through co-occurrence network and bibliometric analysis. The last research objective discusses the future avenues for prospective stakeholders of the hospitality and tourism industry and suggests solutions for unpredictable future situations. All and all, this study contributes to the emerging literature on post-Covid-19 management strategies related to the hospitality and tourism industry through a comprehensive analysis of the extant literature and suggest research gaps that future scholars must address to validate the proposed solutions.

**FIGURE 1:**
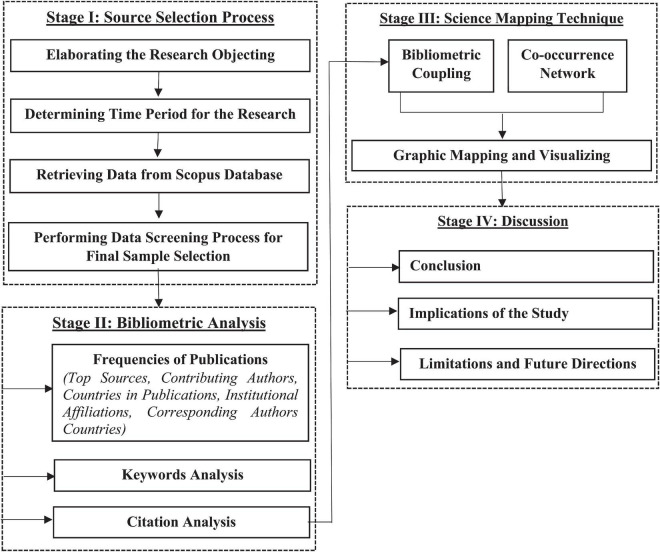
Flow of research.

The remaining manuscript is arranged in four sections. In the first stage, the source selection process is defined; in stage 2, bibliometric analysis is conducted; stage 3 explains the science mapping technique; finally, stage 4 includes the discussion on results, followed by the study implications, limitations, and future directions. Further, the information about the flow of research is elaborated in Figure 1.

## Materials and Methods-A Bibliometric Analysis

Bibliometric analysis is a tool for extracting scholarly information (articles, books, book chapters, conference proceedings, and reports) from well-reputed databases (Google Scholar, Web of Sciences, Scopus, Emerald, Elsevier) (Nasir et al., 2021). The related data is then arranged in chronological order and analyzed to devise future strategies for the study topic. The current study has focused on the post-Covid-19 management strategies for the hospitality and tourism industry; therefore, the basic statistics retrieved from the Scopus database are restricted to January 1, 2020, to July 28 2021. The study focused on the data of the Scopus database due to the limited access to the other databases. We used “bibliometrix 3.0” package of *r*-studio to analyze various aspects of the extracted data related to the topic under study. The query consultation included: TITLE-ABS-KEY {(management OR organization OR organizational) AND (strategies OR policies) AND (hospitality OR tourism OR “Hospitality industry” OR “tourism industry”) AND [(post OR after) AND (covid OR sars OR pandemic OR corona^∗^)]} AND [LIMIT-TO (pub year, 2021) OR LIMIT-TO (pub year, 2020)]. However, the results may likely change with the increased publications related to the post-Covid-19 management strategies for the hospitality and tourism industry in future.

**FIGURE 2:**
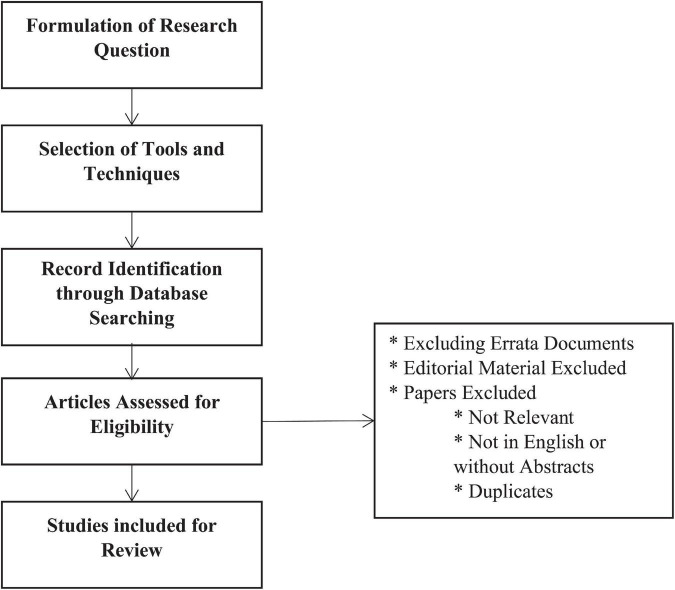
Sample selection criteria.

This research aims to establish a conceptual framework for the prospective scholars that can lead them to explore the new research areas for the post-Covid-19 policies for the hospitality and tourism industry by giving credit to the prior researches that contribute to generate future knowledge in the field. For this purpose, firstly, an extensive literature review is conducted within the scope of the topic under study. Later on, strict criteria for selecting the final data are followed so that only relevant information is included for the analysis. The sorted information can help to improve the understanding of the researchers, practitioners, and policymakers of the management strategies in the post-crisis era. Figure 2 provides detailed information about the sample selection criteria that we adopted for this study.

Initially, we extracted 121 documents, of which 63 were duplicated, irrelevant topics and, therefore, deleted them before final analysis. We ended up with 58 documents from different sources (journals, books, and conference papers, etc.). The current study also included the documents (books, journals, reports, conference papers, letters, notes, and reviews) written in the “English” language. Table 1 provides the information about the description and details of the data used in this study. Total 228 authors make a scientific contribution in the field, out of which ten documents are single-authored while 218 are multi-authored documents. The average citation per document is 4.793, document per author is 0.254, while author and co-author per document are 3.93 and 4.12, respectively. Table 1 also shows that most of the documents are comprised of articles (*n* = 47; 81%). However, the percentage also includes conference papers (*n* = 5; 9%); letter (*n* = 2; 3%); note (*n* = 3; 5%); and review (*n* = 1; 2%).

**TABLE 1:** Description and distribution of main information.

Description	Results
Documents	58
Sources (Journals and Books, etc.)	39
Keywords plus (ID)	413
Author’s keywords (DE)	268
Period	2020–2021
Average citations per document	4.793
Authors	228
Author appearances	239
Authors of single-authored documents	10
Authors of multi-authored documents	218
Documents per author	0.254
Authors per document	3.93
Co-authors per documents	4.12
Collaboration index	4.54
**Document types**	
Article	47
Conference paper	5
Letter	2
Note	3
Review	1

## Results

The bibliometric analysis aims to develop the framework that can assist hospitality and tourism industry managers to design their post-Covid-19 business strategies. It also helps the researchers to delineate current findings for creating future knowledge. For this purpose, firstly, a systematic literature review is conducted to identify the top sources, journals, scholars, corresponding authors, countries, searching keywords, and citations. This sorted information provides a brief introduction about the core contributors in the field. In the second phase of the study, the researchers aim to identify the key themes and streams through the science mapping tools of the conceptual structure and by following a holistic keywords approach as input data. These approaches help to provide an in-depth understanding of the data and suggest a future research agenda to the prospective researchers in the field of hospitality and tourism management.

### Scientific Performance

In the first stage, the scientific performance of the core contributors (sources, journals, scholars, corresponding authors, countries, searching keywords, and citations) is identified.

#### Top Sources

We use Bradford law and source impact to find the top journals publishing the literature relevant to post Covid-19 management strategies for the hospitality and tourism industry. Table 2 shows the journal ranking according to Bradford law which divided the core journals into two main Zones. Zone 1 includes 20 core sources publishing the scientific knowledge related to the topic, while the rest of the publications fall under zone 2. Table 3 describes the source impacts of the journals based on h, g, m-index, TC (total citations), NP (net production), and PY-start (publication starting year). Although scholars did not consider h-index, g-index, and m-index as an accurate predictor of the quality of publications, they still use them in the bibliometric analysis as they believe it somehow provides a general idea about the quality of the research in the field (Merigó and Yang, 2017). The author ranked the articles on the basis of h-index; this measure also undertake total citation and net production while ranking the journal. The values of h, g, m-index (3,4,1.5), and NP (12) is highest for *Sustainability* in 2020, respectively. Whereas Resources, Conservation and Recycling received the highest citations (49) in 2021.

**TABLE 2:** Journal ranking based on Bradford law.

Journals name	Rank	Freq	Cum-freq	Zone
Sustainability (Switzerland)	1	12	12	Zone 1
Worldwide hospitality and tourism themes	2	4	16	Zone 1
Current issues in tourism	3	2	18	Zone 1
International journal of contemporary hospitality management	4	2	20	Zone 1
Resources, conservation and recycling	5	2	22	Zone 2
Smart innovation, systems and technologies	6	2	24	Zone 2
Tourism economics	7	2	26	Zone 2
Acta biomedica	8	1	27	Zone 2
African journal of hospitality, tourism and leisure	9	1	28	Zone 2
Asia Pacific journal of marketing and logistics	10	1	29	Zone 2

**TABLE 3:** Top 10 journals with source impact.

Source	h-index	g-index	m-index	TC	NP	PY-start
Sustainability (Switzerland)	3	4	1.5	27	12	2020
Current issues in tourism	2	2	1	38	2	2020
International journal of contemporary hospitality management	2	2	2	8	2	2021
Resources, conservation and recycling	2	2	2	49	2	2021
Worldwide hospitality and tourism themes	1	2	0.5	4	4	2020
Tourism economics	1	1	0.5	2	2	2020
ACTA biomedica	1	1	0.5	42	1	2020
African journal of hospitality, tourism and leisure	1	1	1	1	1	2021
Forests	1	1	0.5	16	1	2020
Hospitality and society	1	1	1	1	1	2021

*Sustainability* is a top journal for publishing the literature regarding post-Covid-19 business strategies and policies. Recently, Pongsakornrungsilp et al. (2021) revisited the existing crisis management theory and explored how effectively it helps handle the Covid-19 impact during the recovery process. Their findings illustrate that brand management is an effective tool for firms to cope up with the crisis. Along with it, communication with the employees builds strong relationships that engaging them during the recovery phase. *Worldwide hospitality and tourism themes* stand at second rank. In his recent publication, Miller (2021) explained the role of unexpected changes, events, and situations in reshaping the environment for tourism. He emphasized integrating resilience in disaster management, recovery, and continuity strategies in the post-Covid-19 hospitality and tourism industries.

The third important source of publication is Current Issues in Tourism. Shao et al. (2021) discussed the post-Covid recovery strategies for the tourism industry and identified four core themes: prophylactic measures, tourism management and development, policy support and departmental management. Yacoub and ElHajjar (2021) explained that the hotel industry was unprepared to deal with the pandemic. But in the post-Covid period, they started focusing more on safety measures, targeting local tourists, making flexible policies for customer retention, and adopting technologically advanced artificial intelligence techniques. Ibn-Mohammed et al. (2021) emphasize visiting the global economic growth models under the pandemic and introducing new sustainable development strategies. Sousa et al. (2021) highlighted the role of film tourism for market segmentation and attracting tourists in the post-Covid period.

Poretti and Heo (2021) analyzed the stock market reaction of the international hospitality firms after the declaration of Covid-19 as a pandemic by WHO (world health organization). Their analysis indicated that investors overestimated the risk in the short run due to panic and uncertain situations. But later, they started following the asset-light strategy that lessens the negative effect (Bartis et al., 2021) of abnormal returns and operating leverage. The asset-light approach helps to minimize the risk factor as investors started viewing the pandemic situation more rationally, revisiting their investment portfolios, and revising their strategies that somehow helps to reduce information asymmetry. Capolongo et al. (2020) redefined the concept of public health and suggested actions that promote a healthy environment for transforming the existing structure. Bartis et al. (2021) stated that organizations adopted innovative measures like arranging virtual events, using artificial intelligence techniques in business models and training their employees in a post-Covid recovery period.

#### Top Contributing Authors

Table 4 provides information about the prominent contributing authors in the field of hospitality and tourism industry. The data is sorted based on h, g, m-index, TC (total citations), NP (net production), and PY-start (publication starting year). All the articles included in the list were published in 2020 and have the same net production (NP = 2). The first two authors have the same h, g, m-index, and total citations (h-index = 2; g-index = 2; m-index = 1; TC = 13). Del Buono et al. (2020) are one of the leading authors who discussed the issue of over-tourism that most historic European cities face through a shared learning method. They had selected five historical countries and identified the key impediments in the promotion of tourism sectors. According to their findings, the primary issue is the affordable housing facility, and for that, supportive housing policies are needed to be developed at the international and local levels. They also emphasized the imposition of restrictions at the global or regional level in the form of tourist tax to control the extra inflow of the tourists and provide better facilities. Further, they suggested building new hotels and emphasize banning illegal accommodation.

**TABLE 4:** Top 10 authors with source impact.

Author	h_index	g_index	m_index	TC	NP	PY_start
Dastgerdi AS	2	2	1	13	2	2020
de Luca G	2	2	1	13	2	2020
Dimitrakopoulos PG	1	2	0.5	16	2	2020
Francini C	2	2	1	13	2	2020
Gkoumas V	1	2	0.5	16	2	2020
Holtvoeth J	1	2	0.5	16	2	2020
Jones A	1	2	0.5	16	2	2020
Jones N	1	2	0.5	16	2	2020
Kontoleon A	1	2	0.5	16	2	2020
Liu Y	1	1	0.5	1	2	2020

Luca et al. (2020) are amongst the second prominent authors who worked on the common management challenges that historic European cities face due to over-tourism. Their study emphasized revising the monitoring tactics, accommodation policies, and promotional strategies. Dimitrakopoulos has higher total citations than Dastgerdi and de Luca. The h, g, m-index, and total citations of his article are: h-index = 1; g-index = 2; m-index = 0.5; TC = 16. McGinlay et al. (2020) discussed the challenges and consequences of Covid-19 for protected areas that promote sustainable tourism models. Their results stated that overcrowding, the arrival of new visitors, behavioral conflicts are the major challenges. They also emphasized adopting safety measures, introducing information campaigns, establishing one-way paths for entry and exit to deal with the breakdown of the virus. Further, they suggested adopting long-term measures instead of focusing on short-term measures solutions.

In line with the previous study by McGinlay et al. (2020), in which Jones is also a co-author, Jones et al. (2021) suggested in his latest article to develop a mobile app for tourist guidance to avoid overcrowdedness in the protective areas. Many countries started lowering their fees for state-owned attractions to promote local tourism, but the same policy can impact their financial side. Liu J. Y. et al. (2021) proposed a “resource function transformation cost” policy to explain the relationship between the new business model and cost structure. Wen et al. (2021) suggested three strategies to boost up the tourism industry: (1) provide medical facilities to tourists during and after the pandemic phase; (2) improve destination image by promoting China as a globally safe place for travelers; (3) improve the role of traditional China medicine policy for economic recovery.

#### Top Countries in Publications

Table 5 shows the top 10 countries in terms of total publications. China is the country that ranked top in the list of total publications (TP = 26), followed by Spain and the United Kingdom with 19 and 16 total publications, respectively. These top three countries presented almost 55% of the total publications included in the list of top 10 countries. Portugal (TP = 11) and Italy (TP = 10) stands in fourth and fifth positions with approximately 10 and 9% publications. No other country included in the top 10 list attained the double-figure number in a total publication. However, Australia and Indonesia ranked in ninth and tenth numbers, but both have the same number of publications (TP = 5).

**TABLE 5:** Top countries in publications.

Country	Total publications
China	26
Spain	19
United Kingdom	16
Portugal	11
Italy	10
India	7
Romania	6
United States of America	6
Australia	5
Indonesia	5

#### Top Corresponding Authors’ Countries

Table 6 provides information about the corresponding authors’ countries based on SCP and MCP. SCP refers to the single country publications where a research collaboration between the authors of a single country is done. MCP stands for multiple countries publications, which refer to the collaboration between various countries by the scholars. China stands at the top of the list with nine research publications (SCP = 7; MCP = 2). Spain and Portugal attain the second and third rank in terms of total article publication, and their SCP score is 4 and 3, respectively. Whereas in terms of MCP, the United Kingdom and Greece stand at the second number with an MCP score of 2. Canada has only one publication which is a single country publication.

**TABLE 6:** Most relevant corresponding authors’ countries.

Country	Articles	Freq	SCP	MCP	MCP_Ratio
China	9	0.1915	7	2	0.222
Spain	5	0.1064	4	1	0.2
Portugal	4	0.0851	3	1	0.25
United Kingdom	3	0.0638	1	2	0.667
United States of America	3	0.0638	2	1	0.333
Australia	2	0.0426	1	1	0.5
Greece	2	0.0426	0	2	1
Indonesia	2	0.0426	2	0	0
Italy	2	0.0426	2	0	0
Canada	1	0.0213	1	0	0

#### Top Institutional Affiliations

Table 7 reported the core contributing institutions in the post-Covid management literature. The six institutions accounting for 58% included in the top 10 affiliation list have belonged to Spain and China. Universidad de Sevilla, the University of Mlaga and Rey Juan Carlos University have published eight research articles that accounted for 31%. At the same time, Zhejiang Gongshang University, Children’s Hospital of Fudan University and Hainan Women and Children’s Medical Center belong to China, have a total of seven publications that contributed to 27%. The top six affiliated institutions that existed in Spain, Italy, Dubai, China and Greece have reported the same number of publications (3). While the next four institutions technically ranked at the second position also published the same number of articles (2), belonged to Spain, China, and Australia.

**TABLE 7:** Top affiliations of relevant publications.

Affiliations	Articles
Dubai Health Authority	3
Universidad De Sevilla	3
University of Florence	3
University of Mlaga	3
University of The Aegean	3
Zhejiang Gongshang University	3
Childrens Hospital of Fudan University	2
Edith Cowan University	2
Rey Juan Carlos University	2
Hainan Women and Childrens Medical Center	2

#### Keywords Analysis

The keyword analysis highlights the core concepts used in the main body of the articles. Table 8 presented the precise information about the main keywords (abstract, author, title, and keyword plus) related to post Covid management strategies the hospitality and tourism industry employed from January 1, 2020, to July 28, 2021. Some scholars consider keyword plus more significant in investigating the knowledge structure of the scientific field than authors’ keywords in the bibliometric analysis (Zhang et al., 2016). But the same is less informative in reflecting the main content of the article (Palácios et al., 2021). Similarly, some researchers believe that title keywords are more generic and cannot reflect the central article theme; however, the more common keywords in all sections can best represent the research streams (Nasir et al., 2021).

**TABLE 8:** Keywords analysis.

Abstracts keywords		Authors keywords	
Words	Occurrences	Words	Occurrences
Tourism	185	Covid 19	33
Covid	96	Covid 19 pandemic	6
Study	88	Crisis management	4
Pandemic	64	Pandemic	4
Management	56	Tourism	4
Strategies	50	Content analysis	3
Industry	47	Public health	3
Crisis	46	Sustainable development	3
Paper	41	Biodiversity conservation	2
Health	40	China	2

**Title keywords**		**Keyword plus**	

Covid	37	Tourism	13
Tourism	28	Covid 19	11
Pandemic	10	Public health	9
Case	9	Sustainable development	9
Management	9	Tourism management	9
Post covid	8	Pandemic	8
Analysis	6	Strategic approach	8
Strategies	6	Viral disease	8
Crisis	5	Coronavirus disease 2019	7
Development	5	Human	7

**FIGURE 3:**
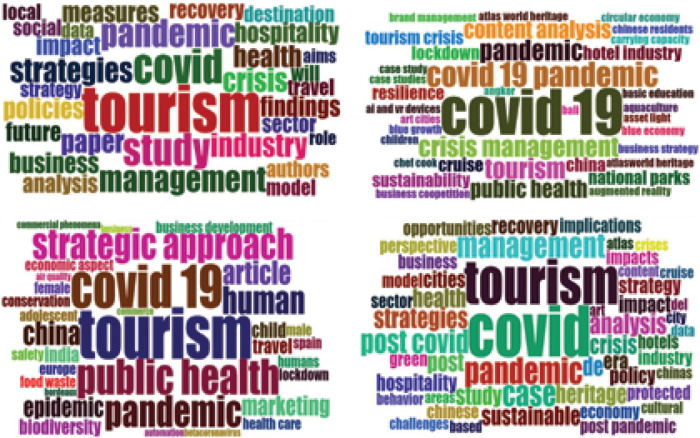
Keyword analysis.

In all the sections, “Covid-19” remains the most commonly used keyword. As the WHO declared Covid-19 a pandemic in 2019, therefore, “pandemic” and “viral disease” are also found in all categories of keywords. This virus is originated in China, so “China” is also included in the author’s keyword. The major consequences of Covid-19 are related to public health and crisis in all most all the segments of the economy. The same is reflected by the keywords of “crisis,” “health” or “public health.” The current study specifically focused on the post-crisis pandemic period strategies in the hospitality and tourism industry. So, the keywords like “industry,” “tourism,” “strategies,” “post Covid,” “management” are also found in the selected documents. Figure 3 presented the four tag clouds to overview the core concepts and terms quickly.

#### Citation Analysis

This section highlights the leading publications based on total citations and citations per year related to the management policies and strategies for the hospitality and tourism industry after the Covid-19 breakout (see Table 9). Ibn-Mohammed et al. (2021) received the highest total citations (TC = 42) and total citations per year (TCPY = 42) on his article published in 2021. They focused on introducing new management policies to promote the “sustainability” concept in future. Capolongo et al. (2020) are the authors of one notable publication that received the second-highest citations (TC = 42; TCPY = 21). They emphasized redefining the concept of public health and suggested the management should implement and promote the same for the survival of their industries. Chen H. et al. (2020) studied the effect of news coverage on the industrial growth of the hospitality and tourism industry during the Covid-19. They identified nine themes through content analysis that helped the management to develop their policies in the post-Covid era.

**TABLE 9:** Top publications with citations.

First authors	Year	Journal	Total citations	TC per Year
Ibn-Mohammed T	2021	Resour Conserv Recycl	42	42
Capolongo S	2020	Acta Biomed	42	21
Chen H	2020	Curr Issues Tour	33	16.5
Renaud L	2020	Tour Geogr	28	14
Yeh SS	2021	Tour Recreat Res	18	18
Mcginlay J	2020	Forests	16	8
Tanrvermi H	2020	J Urban Manag	10	5
de Luca G	2020	Sustainability	10	5
Lee CC	2021	Int J Tour Res	8	8
Chen T	2020	Risk Manage Healthy Policy	8	4

Renaud (2020) reported that industries should promote their local tourism to avoid economic setbacks. Due to pandemic situations, the hospitality and tourism industry is affected mainly due to their reliance on foreign travelers. So, by promoting local tourism, their financial losses can be minimized. They also stressed controlling over-tourism and promote a healthy environment. Yeh (2021) believe that disaster management techniques can be a helpful tool to mitigate the effect of post-crisis. The findings of his study considered open communication with stakeholders a key to building up trust and government-sponsored loans to survive the crisis. McGinlay et al. (2020) suggested a tourism model to control over-crowdedness, behavioral conflicts and hustle at the state-owned attractions. Tanrıvermiş (2020) stated that government should promote a hygienic environment as it is necessary for the survival of the tourism and hospitality industry.

Luca et al. (2020) emphasized revising the monitoring tactics, accommodation policies, and promotional strategies to overcome the over-tourism challenge. Lee et al. (2021) analyzed the influence of geopolitical risk on the global tourism demand. Their findings confirmed the effect of geopolitical risk on the tourist intention to visit the local and international tourist destinations and the overall economic performance of the industry. They also suggested that the organizations should establish crisis management plans to save themselves from inconvenience. They follow the aggressive strategies in a post-crisis period to quickly recover from it. Chen T. et al. (2020) studied the perceived effect of the Chinese government promotional policies on developing the tourism industry. Their findings indicated that from a single perspective, safety policy is considered the best. However, from a general perspective, a combination of safety plus economic policy is better to protect public health and accelerate financial well-being.

Table 10 identified the top 10 countries in terms of total citations. China is the country that ranked top in the list of total citations (TC = 59), followed by Italy (TC = 52) and the United Kingdom (TC = 49), representing 25, 22, and 21%. Taiwan and Greece occupy the fourth and fifth positions with (TC = 18) and (TC = 17). However, in terms of average article citations, Italy ranked at the top position (AAC = 26), followed by Taiwan (AAC = 18) and United Kingdom (AAC = 16.33). Turkey (AAC = 10) and Greece (AAC = 8.50) places at fourth and fifth positions. Lebanon appears at the tenth position in terms of total citations (TC = 3) and average article citation (AAC = 3).

**TABLE 10:** Top countries/regions in citations.

Country/regions	Total citations	Average article citations
China	59	6.56
Italy	52	26.00
United Kingdom	49	16.33
Taiwan	18	18.00
Greece	17	8.50
Spain	15	3.00
Turkey	10	10.00
Portugal	5	1.25
India	4	4.00
Lebanon	3	3.00

## Conceptual Framework

We are considering devising specific underlying theories, themes, and streams under which management strategies for the tourism and hospitality industry are converging. We have devised the bibliometric coupling and co-occurrence network to identify post-pandemic theories, themes, and streams of management strategies.

### Bibliometric Coupling

**FIGURE 4:**
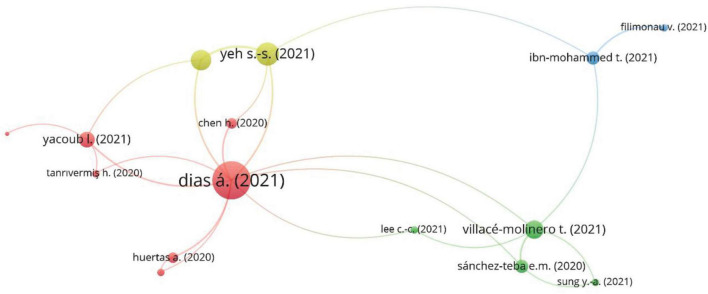
Bibliometric coupling.

From 58 scholarly publications on the underlying topic, it was cumbersome to identify the two or more papers with duplicate citations. Bibliometric coupling checks the similarity between two documents. It occurs when two documents cite the same references (Kessler, 1963). There can be more than one common reference between documents. One article can share one or more references with more than one article that indicates the document’s relative link strength. There are fifty-eight articles to connect on the basics of link strength. We have conducted bibliometric coupling on various conditions. Articles with a minimum of three common references are taken as the basis. Figure 4 represents the bibliometric coupling which highlighted four clusters based on the linkage between the article. The size of the bubble represents the link strength with the association strength normalization method and one thousand iterations. Cluster iteration was set at 10 iterations with one minimum cluster. after these attributes, we have narrowed down four clusters representing various dynamic themes.

Table 11 is the detailed representation of Figure 4 as it shows the labels used in Figure 4. The second column is the title of the articles; column three represents the associated cluster. The red color in Figure 4 represents cluster 1, the green color is cluster 2, the blue color is cluster 3, and the yellow color of Figure 4 is represented by cluster 4. All articles in clusters are linked together based on a specific theme. The fourth and fifth columns are links and total link strength. It represents the connection between the documents. The last column is the number of citations of a specific article.

**TABLE 11:** Publication clustering from bibliometric coupling.

Label	Title	Cluster	Links	Total link strength	Citations
Dias et al., 2021	*“Post-pandemic recovery strategies: Revitalizing lifestyle entrepreneurship”* (Dias et al., 2021)	1	10	15	3
Yacoub and ElHajjar, 2021	*“How do hotels in developing countries manage the impact of Covid-19? The case of Lebanese hotels”* (Yacoub and ElHajjar, 2021)	1	4	5	3
Chen H. et al., 2020	*“A content analysis of Chinese news coverage on Covid-19 and tourism”* (Chen H. et al., 2020)	1	2	3	33
Huertas et al., 2020	*“Crisis communication management by the national tourist organizations of Spain and Italy in the face of Covid-19”* (Huertas et al., 2020)	1	2	3	3
Tanrıvermiş, 2020	*“Possible impacts of Covid-19 outbreak on real estate sector and possible changes to adopt: A situation analysis and general assessment on Turkish perspective”* (Huertas et al., 2020)	1	2	2	10
Shao, 2021	*“What is the policy focus for tourism recovery after the outbreak of Covid-19? A co-word analysis”* (Shao et al., 2021)	1	2	2	5
Mohanty, 2020	*“Augmented reality for relaunching tourism post-Covid-19: socially distant, virtually connected”* (Mohanty et al., 2020)	1	1	1	4
Villacé-Molinero, 2021	*“Understanding the new post-Covid-19 risk scenario: outlooks and challenges for a new era of tourism”* (Villacé-Molinero et al., 2021)	2	5	6	5
Sánchez-Teba, 2020	*“The application of the inbound marketing strategy on costa del sol planning & tourism board. lessons for post-Covid-19 revival”* (Sánchez-Teba et al., 2020)	2	3	4	5
Lee et al., 2021	*“Geopolitical risk and tourism: Evidence from dynamic heterogeneous panel models”* (Lee et al., 2021)	2	2	2	8
Sung et al., 2021	*“Big data analysis of Korean travelers’ behavior in the post-Covid-19 era”* (Sung et al., 2021)	2	2	2	4
Ibn-Mohammed et al., 2021	*“A critical review of the impacts of Covid-19 on the global economy and ecosystems and opportunities for circular economy strategies”* (Ibn-Mohammed et al., 2021)	3	3	4	42
Filimonau, 2021	*“The prospects of waste management in the hospitality sector post Covid-19”* (Filimonau, 2021)	3	1	2	7
Yeh, 2021	*“Tourism recovery strategy against Covid-19 pandemic”* (Yeh, 2021)	4	4	8	18
Liu J. Y. et al., 2021	*“Taking a break is for accomplishing a longer journey: Hospitality industry in Macao under the Covid-19 pandemic”* (Liu M. T. et al., 2021)	4	3	7	5

Cluster 1, shown in red color in Figure 4, presents the post-Covid recovery of the tourism sector. Dias et al. (2021) is the most related article in the mix and explored the post-pandemic recovery strategies for tourism lifestyle entrepreneurs. They suggest that to overcome the post crises situation, there should enhance communication capacity, availability of niche markets for small scale entrepreneurs, and impediment the common limitations of tourism lifestyle entrepreneurs. Yacoub and ElHajjar (2021) conducted interviews of 4 and 5-star hotels to analyze the post-pandemic crisis management measures. Chen H. et al. (2020) is the most cited article in cluster one. They provided insight for the post-pandemic investigation of the tourism crisis. They study the post-pandemic tourism control and activities, development of cultural venues, hospitality industry role and management strategies, national command, control and local response, corporate self-assessment and development strategy, tourism disputes and solutions, post-crisis tourism product, people sentiments, and government assistance. Technological change strategy is required to improve these themes after the pandemic.

Cluster 2 in green is more related to post-pandemic risk factors of the tourism and hospitality industry. With total link strength of 6, Villacé-Molinero et al. (2021) propose various travel risk scenarios during the pandemic and suggested measures to improve travelers’ confidence after the crisis. Sánchez-Teba et al. (2020) discussed the reduced risk of tourism after the pandemic. They proposed a sustainable tourist relationship model implemented by Costa Del Sol after the pandemic to attract tourists. Lee et al. (2021) analyzed the impact of geographical risk factors on tourism. They checked the causal effect of geographical risk with tourism with moderating effect of a pandemic outbreak. They found the significant negative impact of geographical risks on tourism, and with the moderation of the outbreak, the significance of this negative relationship was enhanced. Furthermore, there is certain development in tourism and traveling, such as tourists prefer domestic venues for traveling and prefer eco-trips (Sung et al., 2021).

Blue cluster number 3 is related to the ecosystem and economic strategies. This cluster possess the article with the highest number of citations. Ibn-Mohammed et al. (2021) provided a critical review of the economic effects of the pandemic and suggested strategies toward an efficient low carbon economy. They directed a more sustainable economic system with energy gulping manufacturing procedures and recommended sector-specific low environmental harm strategies to create a sustainable post-Covid world. Filimonau (2021) further discussed the socio-economic perspective of SARS-COV 2. With the collapse of the hospitality industry during the pandemic, his manuscript focused on developing strategies to aid against the hospitality sector’s food and plastic waste problem in the post-pandemic world. They suggested green innovation for plastic waste management and discussed institutional pre-requisites for effective implementations of waste management strategies.

Cluster 4 is close to cluster 1, and it is shown in yellow (Figure 4). This cluster is related to crises and disaster management for the survival of the tourism industry (Yeh, 2021). This cluster highlights the coexistence of opportunities and challenges from the experience of epidemic. It suggests the practice and policies that are effective during a pandemic (Liu M. T. et al., 2021).

### Co-occurrence Network

A co-occurrence network is the holistic keyword analysis to identify patterns of various themes in literature. This study provides various themes that can be used as the future directions for developing the management strategies of the hospitality and tourism industry. It helps in reviving the industry and provides directions for more profitability and a systematically developed industrial base.

**FIGURE 5:**
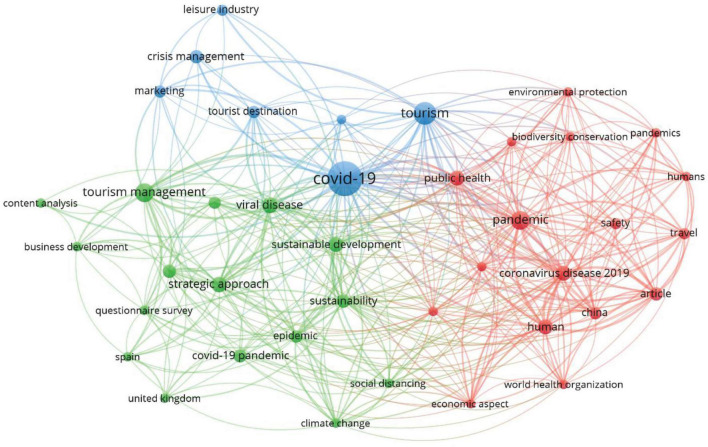
Co-occurrence network analysis.

While using Vosviewer, we have developed a co-occurrence analysis based on keywords with parameters set to normalization method of association strength with a maximum of one thousand iterations, step size convergence of 0.001 and reduction of 0.75. We have set the minimum cluster to one with a clustering resolution of 0.90, and association weights are based on keyword occurrences Figure 5 depicts the co-occurrence network built based on links and occurrences of the specific keyword. From the depiction, we have highlighted the main keywords in separate Table 12 to provide meaning research stream for every cluster of keywords. The keywords are arranged according to their number of links with other keywords and occurrence in literature.

**TABLE 12:** Holistic co-occurrence network with research streams.

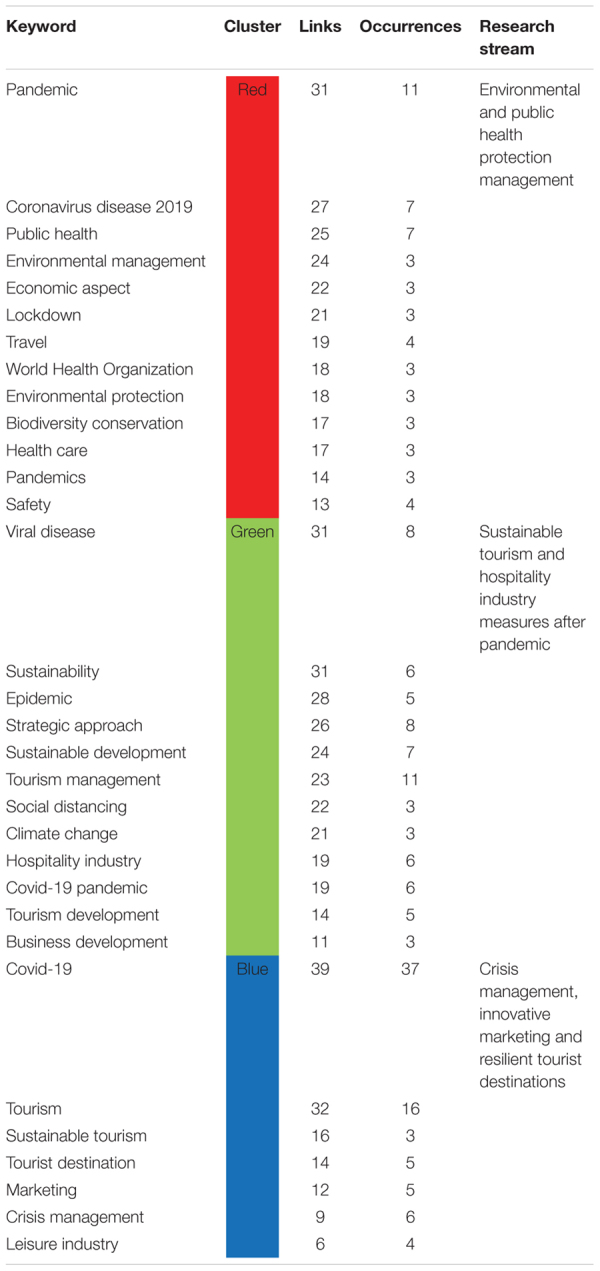

The red cluster is the largest, and it represents the keywords that represent the research stream of environmental and public health protection management strategies. As a result of pandemics and the implementation of lockdowns in many countries, most industrial activities were halted because of public health concerns. For industrial survival, eco-protection regulations are required (Patterson Edward et al., 2021). As most countries are going through the third wave of pandemics, it is important to specify certain protected areas where people can safely visit under some protected management strategies. McGinlay et al. (2020) suggested some tourism and hospitality public protection management strategies such as educational campaigns, avoiding overcrowding by managing tourist numbers, targeting new profiles of visitors, and sustainable market models of tourism.

Green cluster is related to strategic and sustainable tourism and hospitality industry measures after the pandemic. Government policies play a significant role in developing the resilient future of tourism and the hospitality industry with new business design and working capital availability (Madeira et al., 2021). Digital entrepreneurship can also achieve many goals of eco-friendly traveling, hospitality and marketing (Sánchez-Teba et al., 2020). Another crucial factor is implementing green practices in the hospitality industry to curb health and related environmental issues. Green motives and green business strategies are imperative measures for the sustainable development of the hospitality industry (Yousaf et al., 2021). The tendency of post-Covid recovery is directly related to the capacity to create strategies and policies that take advantage of natural resources and convert it into a socio-economic development opportunity for tourism and hospitality (Mestanza-Ramón and Jiménez-Caballero, 2021).

The research stream of the blue cluster is suggested to be crisis management, innovative marketing and resilient tourism destinations. Post pandemic tourism development is dependent on dynamic inbound tourism programs (Wen et al., 2021). Government, tourism, and hospitality-related initiative and practices are needed to develop strategies to promote practices such as health and hygiene, workforce and training for crisis management, booking flexibility, dynamic cancelation policies, community support and digital contracts (Salem et al., 2021).

## Conclusion

Although the rapid increase in scholarly publications addresses the post-Covid management strategies for the hospitality and tourism industry, it is surprising that no research has yet created the synthesis in the area. The current study conducts a bibliometric analysis to report the core contributors (journals, authors, countries, affiliation, and corresponding authors, etc.) in the field. It also constitutes the citations, keyword, and co-occurrence analysis to identify the main concepts and research streams for the future solutions of the industry. Data were extracted from the Scopus database for the period of January 1, 2020, to July 28, 2021. After defining the selection criteria and removing the duplicates, the final sample ended up at 58 scholarly documents, taken from 39 sources, covering 239 authors appearances. “Sustainability” is the major journal that has grown the most over the years. China is one of the main contributing countries that publish the most significant articles that received the highest citations and also ranked at the top in the correspondence. Spain and China are the countries that reported the highest institutional affiliation.

The study reports two methods under a conceptual framework and suggests four research themes and three future research streams regarding post-Covidmanagement strategies of the hospitality and tourism industry. The first method this study report is the bibliometric coupling that divides the post-Covid management strategies literature into four clusters. Each cluster represents a theme. The first theme is “post-Covid recovery of tourism sector” which represents various management strategies that needed to be altered for recovery after the pandemic. Furthermore, cluster 2 suggests a theme titled “post-pandemic risk factors of tourism and hospitality industry”. This theme discusses various risk factors affecting the development of the tourism and hospitality industry and proposes management measures to curb these uncertainties. The third theme is related to “ecosystem and economic strategies” which suggests post-Covid management strategies for environmental and macroeconomy challenges. Cluster 4 represents the theme that deals with crises and disaster management. The study deploys a co-occurrence network to provide three favorable research streams for post-Covid management strategies for the tourism and hospitality industry. First, researchers and academicians can take the route of environmental and public health protection management under post-Covid management strategies of the tourism and hospitality industry. Secord research stream is related to Sustainable Tourism and hospitality industry measures after the pandemic. The third direction for post-Covid management strategies is crisis management, innovative marketing and resilient tourist destinations.

### Implications of the Study

Theoretically, this research serves as a foundation for future studies in the hospitality and tourism industry by providing an avenue to understand the development of a new paradigm and modification of the existing theories under the disruption caused by Covid-19. The research streams identified in this study can help the scholars to devise post-Covid management strategies and can contribute to the emergence body of knowledge in the field. Second, the current study is beneficial for the hospitality and tourism industry management, who are more anxious to adopt a proactive approach to understand which policies, strategies, and practices are required to survive in an unprecedented situation. The Covid-19 crisis is a new and complex scenario for the organizations that give a practical lesson to the managers to be the forward thinker. The firms can gain even a competitive advantage if they restructure their existing business models according to the transition of the economy, society, and technology. Third, it will guide the managers to apply advanced technologies, artificial intelligence tools and modern disaster management practices as coping up strategies that will benefit them to achieve sustainable development. Fourth, it will help the policymakers to introduce new policies and identify the potential changes in the existing rules to deal with the major economic issues and restore the shuttered services, like travel, tourism, hospitality.

### Limitations and Future Directions

The present study faces certain limitations that provide guidelines to future scholars to broader the canvas of the research. First, it enriches the current research on management strategies in the epidemic-induced hospitality and tourism industry and the methodological literature by employing a bibliometric and co-occurrence analysis. Further, it invites future researchers to investigate the topics such as stakeholder’s sentiment, promotional strategies during the industry revival phase, application of artificial intelligence techniques, etc., in developing the management strategies. Second, the data is retrieved from the Scopus database, considered one of the most reputable databases. Still, some other databases like Web of Sciences and other non-indexed journals are not considered in the analysis. Future researchers may include the suggested databases to recommend a better solution to the industry. Third, the current study only focuses on the hospitality and tourism industry. Future researchers can also explore the crisis response strategies in other sectors by employing the same methodological technique. It will help to increase scholarly contributions that can potentially change future research trends. Fourth, it ignores the population factor while analyzing the geographic distribution of the publications. The result may be different if the correction effect is implied. Future scholars also consider it in their investigations. Fifth for future direction, managers, policymakers, researchers and academicians can take notes from the conceptual framework of this study. This study suggests research streams and themes which provide literature segregation and established future direction.

## Data Availability Statement

The raw data supporting the conclusions of this article will be made available by the authors, without undue reservation.

## Author Contributions

KK contributed significantly in influential aspects of the article. AN contributed to conceptual aspects of the article. SS contributed to structure, implications, future directions and review of the article. All authors contributed to the article and approved the submitted version.

## Conflict of Interest

The authors declare that the research was conducted in the absence of any commercial or financial relationships that could be construed as a potential conflict of interest.

## Publisher’s Note

All claims expressed in this article are solely those of the authors and do not necessarily represent those of their affiliated organizations, or those of the publisher, the editors and the reviewers. Any product that may be evaluated in this article, or claim that may be made by its manufacturer, is not guaranteed or endorsed by the publisher.
